# Probing the Gelation Synergies and Anti-*Escherichia coli* Activity of Fmoc-Phenylalanine/Graphene Oxide Hybrid Hydrogel

**DOI:** 10.1021/acsomega.2c07700

**Published:** 2023-03-08

**Authors:** Efstratios D. Sitsanidis, Lara A. L. Dutra, Johanna Schirmer, Romain Chevigny, Manu Lahtinen, Andreas Johansson, Carmen C. Piras, David K. Smith, Marja Tiirola, Mika Pettersson, Maija Nissinen

**Affiliations:** † Department of Chemistry, Nanoscience Center, University of Jyväskylä, P.O. Box 35, FI-40014 Jyväskylä, Finland; ‡ Department of Biological and Environmental Sciences, Nanoscience Center, University of Jyväskylä, P.O. Box 35, FI-40014 Jyväskylä, Finland; § Department of Physics, Nanoscience Center, University of Jyväskylä, P.O. Box 35, FI-40014 Jyväskylä, Finland; ∥ Department of Chemistry, University of York, Heslington, York, YO10 5DD, United Kingdom

## Abstract

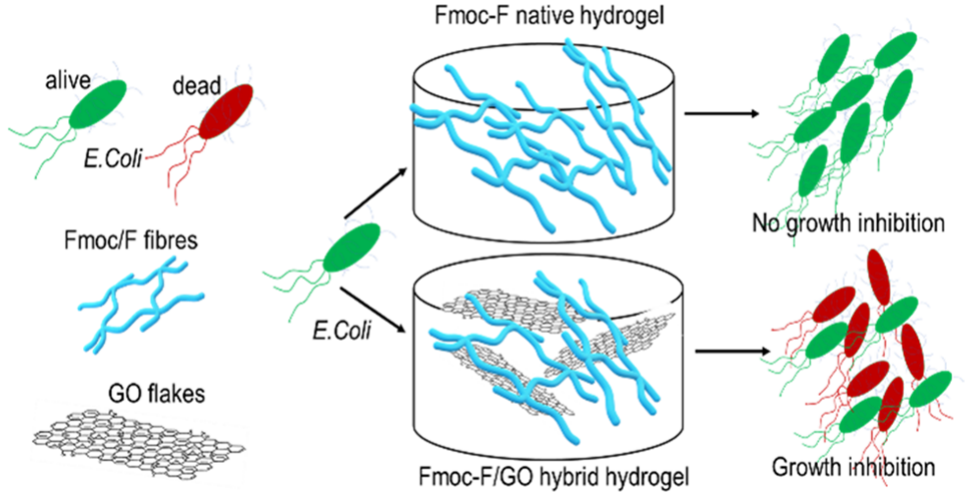

The *N*-fluorenyl-9-methyloxycarbonyl (Fmoc)-protected amino acids have shown high antimicrobial application potential, among which the phenylalanine derivative (Fmoc-F) is the most well-known representative. However, the activity spectrum of Fmoc-F is restricted to Gram-positive bacteria only. The demand for efficient antimicrobial materials expanded research into graphene and its derivatives, although the reported results are somewhat controversial. Herein, we combined graphene oxide (GO) flakes with Fmoc-F amino acid to form Fmoc-F/GO hybrid hydrogel for the first time. We studied the synergistic effect of each component on gelation and assessed the material’s bactericidal activity on Gram-negative *Escherichia coli* (*E. coli*). GO flakes do not affect Fmoc-F self-assembly per se but modulate the elasticity of the gel and speed up its formation. The hybrid hydrogel affects *E. coli* survival, initially causing abrupt bacterial death followed by the recovery of the surviving ones due to the inoculum effect (IE). The combination of graphene with amino acids is a step forward in developing antimicrobial gels due to their easy preparation, chemical modification, graphene functionalization, cost-effectiveness, and physicochemical/biological synergy of each component.

## Introduction

Microbial infections pose a significant threat to human health and are one of the major concerns in public healthcare.^1^ Despite the advances in drug development, limitations associated with the treatment of pathogens include antimicrobial resistance (AMR) toward existing medication and the appearance of new diseases.^2^ Currently, new approaches in antimicrobial therapeutics^3^ and materials are constantly introduced, such as polymers, ceramics, nanoparticles, biomacromolecules, small organic molecules, and hydrogels.^4−7^

Hydrogels have gained momentum for the treatment and prevention of microbial infections due to their physicochemical and viscoelastic properties, cost-effectiveness, ease of preparation, and manufacturing upscale. In addition, they have high water content and combine low toxicity (high biocompatibility toward mammalian cells) with antimicrobial activity. Their activity can be either inherent or, for example, caused by incorporating antimicrobial agents within the gel matrix, which can increase their spectrum of activity.^8,9^ Recently, several amino acid and peptide-based supramolecular gels have been introduced, of which the *N*-fluorenylmethyloxycarbonyl (Fmoc)-protected analogues have shown high application potential.^10−13^

In particular, the phenylalanine derivative (Fmoc-F) has exhibited antibacterial activity against Gram-positive bacteria, both in the solution and gel state, via a mechanism disrupting the bacterial membrane/wall.^14,15^ Additionally, Fmoc-F inhibits the formation of biofilms and eradicates the already formed ones over surfaces due to its surfactant properties.^16^ Despite its efficacy over Gram-positive bacteria, its biocidal effect on Gram-negative bacteria is limited due to its inability to cross the bacterial membrane of Gram-negative microbes. Therefore, to increase the antibacterial spectrum of the amino acid, several Fmoc-F hybrid gels have been fabricated, exploiting the synergistic effect of incorporated antimicrobial agents, for example, aztreonam (AZT) antibiotic,^17^ silver ions,^18^ berberine chloride,^19^ and salicylic acid.^20^

The research for efficient antimicrobial materials has expanded into carbon nanomaterials, such as graphite (Gt), graphite oxide (GtO), graphene oxide (GO), reduced graphene oxide (rGO), carbon nanotubes (CN), and fullerenes.^21,22^ GO forms stable colloids in water and can be easily chemically modified. Therefore, its antimicrobial activity has been extensively studied against Gram-positive/negative pathogens.^23,24^ Graphene-based materials display antibacterial action as they disrupt the cell membrane and induce oxidative stress by producing reactive oxygen species (ROS). However, the reported results are somewhat controversial since their activity is influenced by several factors, such as their size, morphology, purity, concentration, and type of functionalization.^25,26^

Since GO flakes have been reported to show antibacterial activity against Gram-negative *Escherichia coli* (*E. coli*),^21^ their incorporation within the Fmoc-F gel network could expand the antibacterial spectrum of the amino acid against *E. coli*.^14^ In this study, we combined GO flakes with commercially available Fmoc-F amino acid for the first time to produce Fmoc-F/GO hybrid hydrogel. We investigated the synergistic effect of each component on the gelation process spectroscopically and assessed the macro-/microscopic properties of the hybrid material (Fmoc-F/GO) in relation to the native Fmoc-F hydrogel. In addition, we investigated the antimicrobial activity of the formed gel and its components against Gram-negative *E. coli*.

## Materials and Methods

### Materials

*N*-Fluorenyl-9-methoxycarbonyl-l-phenylalanine (Fmoc-F) was purchased from Sigma-Aldrich, GO water dispersion (0.4 wt %) from Graphenea, and rGO powder (98–99%) from Wholesale Graphene. All reagents were used as supplied.

### Preparation of Hydrogels

#### Fmoc-F Native Hydrogel

A suspension of Fmoc-F (2.0 mg/mL) in phosphate buffer solution (PBS, 50 mM, pH 7.4) was sonicated for 2 min and heated at 80 °C for 30 min. The obtained transparent solution was then left to cool down at room temperature for 12 h, giving a self-supporting hydrogel as verified by vial inversion.

#### Fmoc-F/GO Hybrid Hydrogel

GO flakes were formed by drying GO water dispersion (0.4 wt %) under a vacuum for 2 days. The obtained flakes were suspended in PBS solution (50 mM, pH 7.4) at several concentrations (0.2, 0.5, 0.75, 1.0 mg/mL) by sonication (15 min) before the addition of Fmoc-F (2.0 mg/mL). The resulting Fmoc-F/GO suspension was sonicated (2 min) and heated at 80 °C (30 min). Gelation occurred at room temperature after 12 h and was assessed by the vial inversion method. The GO flakes remained equally distributed through the final gel.

### Instrumentation

#### Fluorescence Spectroscopy

Emission spectra were recorded on the Varian Cary Eclipse fluorescence spectrophotometer. Gel samples were formed *in situ* in a quartz cuvette with a path length of 1 cm. The excitation wavelength was 296 nm. Both excitation and emission slit widths were 5 nm.

#### Fourier Transform Infrared (FT-IR) Spectroscopy

IR spectra were measured on Bruker Tensor 27 FT-IR spectrometer in Attenuated Total Reflection (ATR) mode. (Spectral width: 400–4000 cm^–1^; absorption mode; step: 2 cm^–1^; the number of scans: 124). All spectra were baseline corrected.

#### Raman Spectroscopy

Raman spectra were recorded on Bruker Optics SENTERRA R200-785 Raman microscope (Laser 785 nm). Gels were dried under a vacuum for 2 days and placed on a microscope glass slide before measurement.

#### Microscopy

Helium ion microscopy (HIM) images were captured on the Zeiss Orion Nanofab microscope and transmission electron microscopy (TEM) images on the JEOL JEM-1400HC microscope. Atomic force microscopy (AFM) imaging was performed on a Bruker Dimension Icon atomic force microscope using PeakForce tapping mode. ScanAsyst-Air probes from Bruker were used during imaging with the peak force set to 2.0 nN. All AFM images were processed with NanoScope Analysis 1.9 software. To prepare xerogel samples for microscopy imaging, carbon films (400 mesh copper grids, Agar Scientific) were dipped into the gels and allowed to dry in the open air overnight.

#### Rheology

Oscillation rheology was performed on the Malvern Kinexus Pro+ rheometer, fitted with an 8 mm parallel plate upper geometry. All gel samples (1.0 mL volume) were prepared in homemade glass chambers and transferred onto the lower geometry of the instrument as intact gel pellets. Amplitude sweep measurements were performed at an angular frequency of 1.0 Hz, using shear strain (γ%) within the range of 0.05–100% at 25 °C. Frequency sweep measurements were performed in triplicate within the linear viscoelastic region (LVR) where the elastic (*G*′) and loss (*G*″) moduli are independent of the strain amplitude. Each measurement was performed using a shear strain (γ%) of 0.25%, at a range of 0.1 to 100 rad/s at 25 °C.

#### Thermogravimetric Analysis (TGA)

Thermogravimetric analysis was performed on PerkinElmer STA 6000 simultaneous thermogravimetric and differential scanning calorimetric analyzer (TG/DSC). Each sample was placed in an open platinum crucible and heated under air atmosphere (flow rate of 40 mL/min) with a heating rate of 10 °C/min at a temperature range of 20–600 °C. The temperature calibration of the analyzer was based on the melting points of indium (156.60 °C) and zinc (419.5 °C). The weight balance was calibrated at room temperature with a standard weight of 50.0 mg. The used sample weights were 6.0–7.0 mg.

#### Powder X-ray Diffraction (PXRD)

Powder X-ray diffraction measurements were performed on a PANalytical X’Pert PRO MPD diffractometer in Bragg–Brentano geometry using Johansson monochromator generated Cu Kα1 radiation (λ = 1.5406 Å; 45 kV, 40 mA). Each lightly hand-ground powder sample was prepared on a silicon-made “zero-background” inducing holder using petroleum jelly as an adhesive. Diffraction patterns were recorded from a spinning sample by a position-sensitive X’Celerator detector using continuous scanning mode in a 2θ range of 4–70° with a step size of 0.017° and a counting time of 200 s/step. Diffraction data were analyzed using Malvern Panalytical HighScore Plus (v. 4.8).^27^ The unit cell parameters of neat Fmoc-F powder at RT were determined by the Pawley method^28^ using the corresponding single crystal structure parameters (CSD database^29^ entry OGIXOT^30^) as the basis of least-squares refinement. Variable parameters were as follows: zero-offset, polynomial background, sample displacement, unit cell, and peak profile parameters. Refined unit cell parameters were used for monitoring the structural properties of Fmoc-F and Fmoc-F/GO hybrid xerogels.

### Antimicrobial Screening

The antimicrobial activity of Fmoc-F/GO hybrid hydrogel against Gram-negative *E. coli* (strain DSM 882) was assessed by evaluating the bacterial growth/culture density over time (optical density-OD_600_). Two-fold serial dilutions of the Fmoc-F/GO hybrid gel and its corresponding components (Fmoc-F native gel, GO suspension and PBS) were prepared in Luria–Bertani (LB) broth. Fresh *E. coli* culture, in the exponential growth phase, was used to prepare the bacterial inoculum to a final density of 1.5 × 10^6^ CFU/mL in testing samples. The gel samples, prepared at a range of concentrations (Table 1), were pipetted in a honeycomb 96-well plate (200 μL/well) and incubated in a Bioscreen C spectrophotometer (37 °C, continuous shaking with low amplitude and normal speed, OD_600_ readings at 10 min intervals for 24 h). The OD_600_ background values were obtained by subtraction of the negative control values. The OD_600_ background values of the hybrid gel and each component were obtained from samples prepared without bacterial inoculum. The control growth curve for each dilution (with growth medium) was based on the bacterial growth in the presence of the basic growth medium.

**Table 1:** Concentration of Fmoc-F/GO Hybrid Hydrogel Samples for OD_600_ Screening

gel components	D1	D2	D3	D4	D5
Fmoc-F (mg/mL)^a^	1.0	0.5	0.25	0.125	0.0625
GO (μg/mL)^a^	125	62.5	31.25	15.625	7.8125

a Given concentrations of each component of the Fmoc-F/GO hybrid hydrogels prepared by serial dilutions (D1–D5).

Gel samples were prepared based on the given gelation protocol and sterilized under UV light for 1 h. Fluorescence microscopy imaging of the bacteria was performed using a Leica TCS SP8 Falcon microscope. The bacterial cell viability was assessed after 5 h of incubation. *E. coli* were stained by a mixture of SYTO 9 (33.4 μM working solution) and propidium iodide (PI, 400 μM working solution) stains. The obtained images were processed by Fiji2 (ImageJ2) software.

## Results and Discussion

### Gel Fabrication and Morphological Features

The gelation efficacy of the Fmoc-F/GO hybrid system was assessed by a series of concentration screening trials. The critical gelation concentration (CGC) of the protected amino acid (Fmoc-F) for both gels, native and hybrid, was found to be 2.0 mg/mL (Tables S1, S2). For the hybrid material, the gelation outcome depended only on the amino acid concentration, irrespective of the added amount of GO flakes (Table S2). The gel-to-sol phase transition temperature (*T*_gel–sol_) was measured by controlled heating of the gels. The *T*_gel–sol_ of the hybrid hydrogel increased only by increasing the amino acid concentration, while the incorporation of GO flakes at different concentrations showed negligible effects (Tables S3, S4). The *T*_gel–sol_ study verified the thermoreversible nature (gel-to-sol-to-gel) of the hybrid gel system since all test samples reformed upon cooling within 12 h.

**Figure 1:**
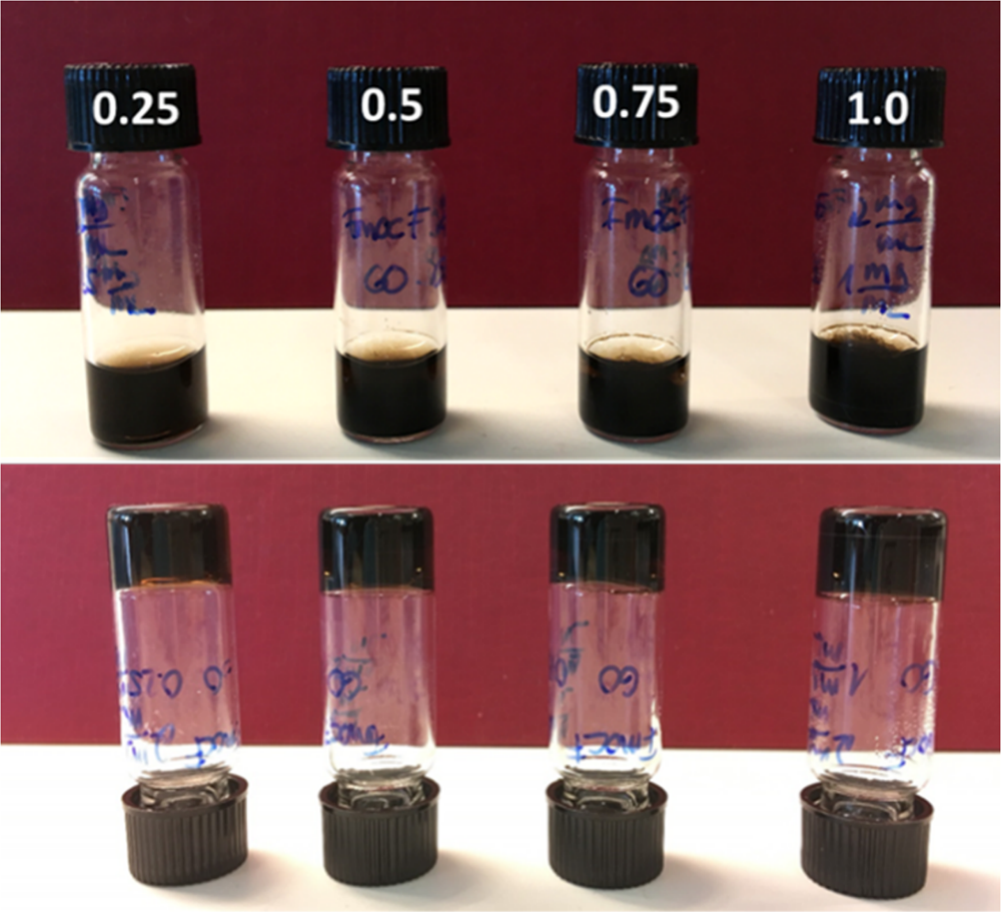
Gelation screening of the Fmoc-F/GO hybrid system at a range of GO concentrations (0.25–1.0 mg/mL). Homogeneous self-supporting gels were formed regardless of the amount of GO. The concentration of Fmoc-F was kept constant (2.0 mg/mL).

Under the given gelation conditions, the suspension of Fmoc-F/GO yielded a homogeneous self-supporting hydrogel, i.e., no phase separation or precipitation of the GO flakes was observed (Figure 1). However, heating the suspension at a higher temperature and for a longer time (95 °C, 1 h) led to the precipitation of GO (Figure S1). When rGO powder was incorporated into the amino acid solution, nonhomogeneous gels formed under the standard gelation conditions (heating at 80 °C for 30 min). Indeed, rGO precipitated in all trials at all used concentrations (0.25–1.0 mg/mL, Figure S2). The increased aggregation of GO at a higher temperature may be attributed to various processes such as enhanced collision frequency, cation dehydration, and reduced electrostatic repulsion, as reported by Gao et al.^31^ in their aggregation kinetics studies of GO in mono- and divalent aqueous solutions.

**Figure 2:**
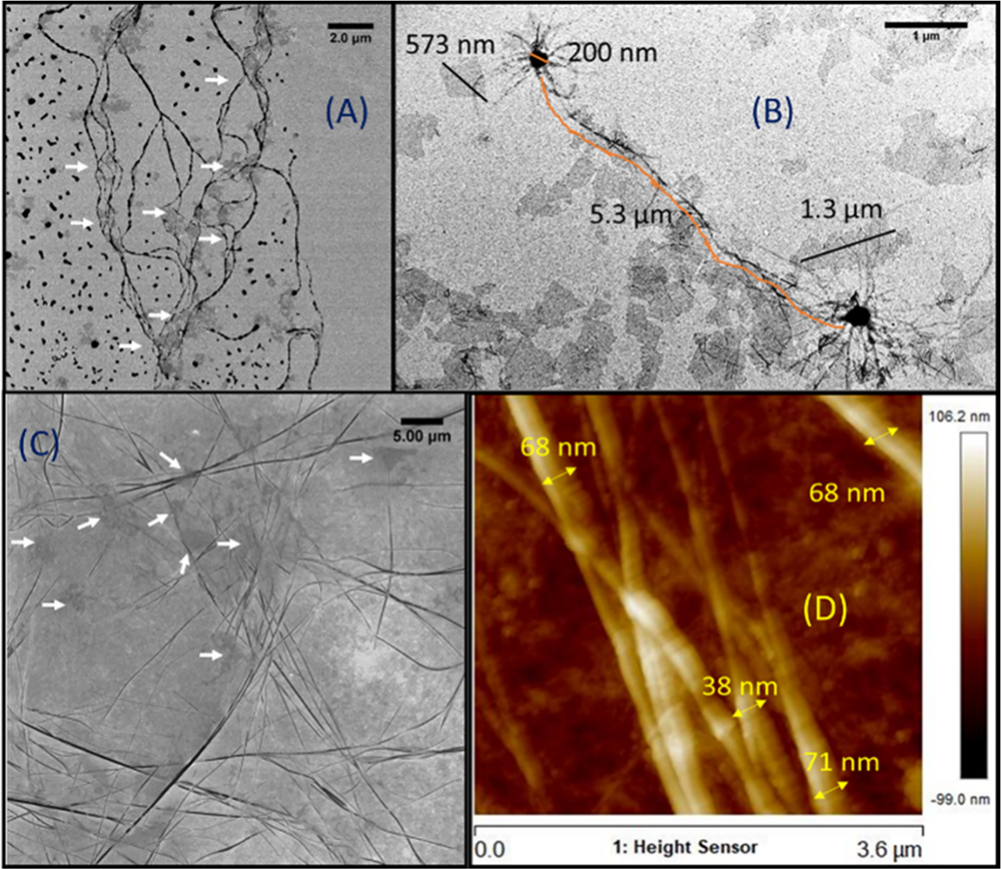
Microscopy images of the Fmoc-F/GO hybrid hydrogel. (A, C) HIM images; (B) TEM image; (D) AFM image with given dimensions of the fibers. The concentrations of Fmoc-F and GO were 2.0 and 0.25 mg/mL, respectively.

The morphology of the hybrid gel network was investigated by HIM, TEM, and AFM. The formed Fmoc-F fibers were similar in shape, width, and length among the native and hybrid materials (Figures 2, S3), suggesting that the presence of GO flakes did not affect the self-assembly of the amino acid. Therefore, the molecular packing of the Fmoc-F building blocks seems to follow a specific hierarchy, initially forming one-dimensional polymeric molecular chains, which lead to higher architectures that interact with GO, *vide infra*. The three-dimensional network comprises single, branched, and entangled fibers and fine fibers in coiled-coil constructions. Their length varies up to several micrometers, and their width is within the range of ∼40–70 nm (Figure 2D).

The GO flakes, seen as semitransparent sheets in the TEM and HIM micrographs, have various dimensions from nano to micrometers. The flakes are well dispersed in the gel network and encircled by Fmoc-F fibrillar loops (Figures 2A,C, white arrows). Indeed, the fibers are formed on the surface, around the edges and between the GO flakes, showcasing the development of noncovalent interactions between the already-formed fibers and GO. GO flakes do not seem to affect the self-assembly per se. However, the size of the flakes needs to be investigated further regarding the nucleation step of Fmoc-F to identify potential connection between the hydrogelation kinetics of the amino acid and the size of GO flakes.

In addition, microscopy imaging revealed spherulitic structures or nucleation points, out of which fibers grow and interpenetrate to adjacent spherulites (Figure 2B). Such structures (microcrystals) have previously been reported in Fmoc-F hydrogels at low pH values, originating from bundles of needle-shaped crystals.^30^ When spin-cast, the structurally similar diphenylalanine (F–F) dipeptide also grows dendritic structures, which have been interpreted as two-dimensional spherulites.^32^ For our hybrid Fmoc-F/GO material, the spherulitic pattern does not cover the entire gel network, which mostly consists of branched, entangled fibers. The hydrogel sample was allowed to dry overnight in the open air before imaging, which might have led to the crystallization of Fmoc-F and the formation of the observed spherulites.

### Mechanical Properties and Thermogravimetric Analysis

**Figure 3:**
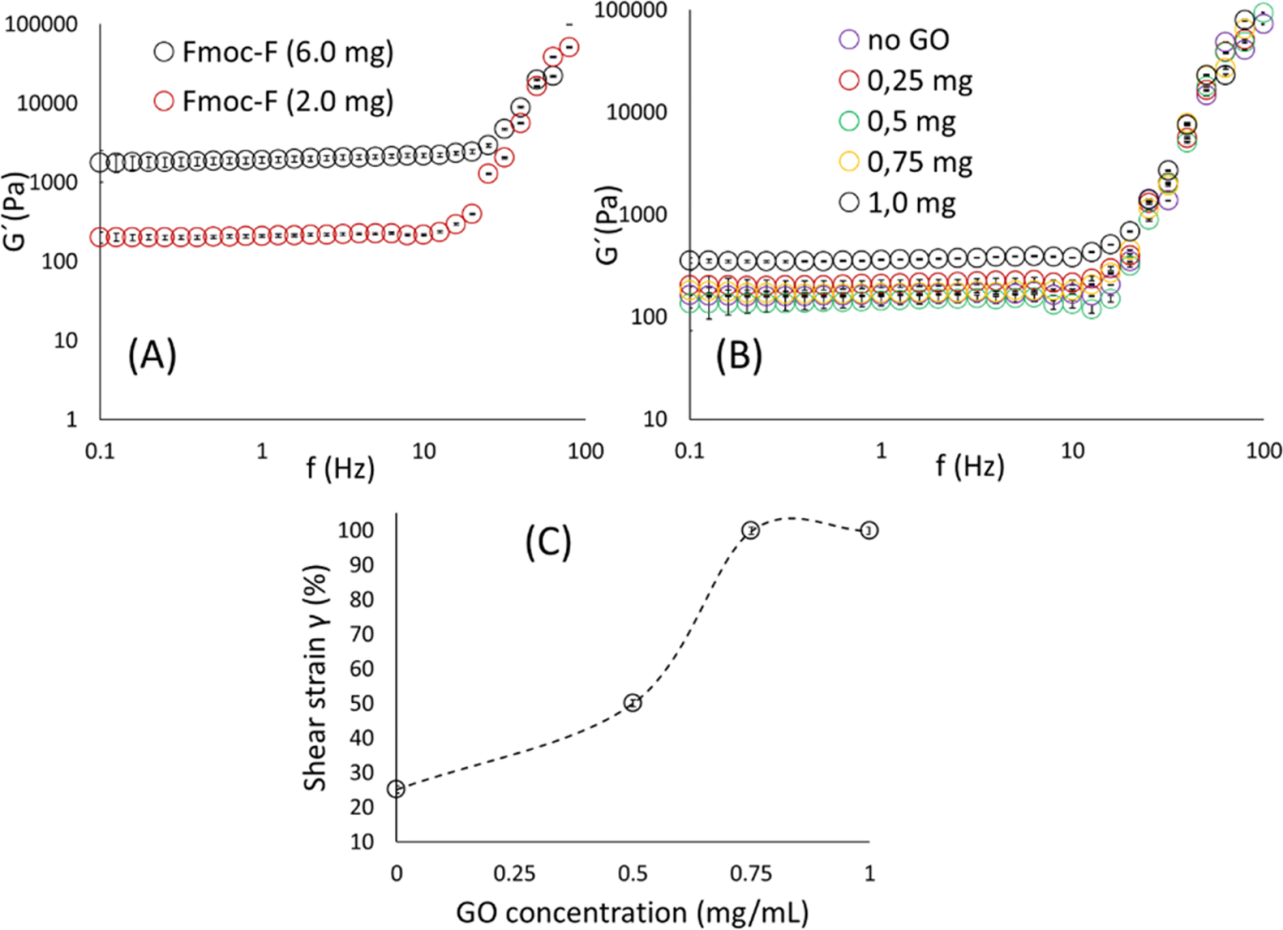
Rheology studies of the hybrid hydrogel. (A) The effect of Fmoc-F concentration on the stiffness (*G*′) of the material. The concentration of GO was kept constant at 0.25 mg/mL. (B) The effect of GO concentration on the stiffness (*G*′) of the material. The concentration of Fmoc-F was kept constant at 2.0 mg/mL. The corresponding *G*″ values are given in Figure S5A,B. (C) Assessing the elasticity of the hybrid gel: Comparing the *G*′ and *G*″ cross points of the amplitude sweep measurements in contrast to the amount of added GO. The concentration of Fmoc-F was kept constant at 2.0 mg/mL. Error bars represent standard deviation.

The viscoelastic properties of the hybrid hydrogel were assessed by oscillatory rheology studies (Figure 3). The frequency sweep measurements were performed on self-supporting gels within the linear viscoelastic region (LVR), in which the storage (*G*′) and loss moduli (*G*″) are independent of the strain amplitude. For both the native and hybrid hydrogels, the *G*′ had a higher value than the *G*′′, confirming the materials’ viscoelastic nature (gel state) (Figures 3, S4, S5). The stiffness of the hybrid material depends only on the amino acid concentration (Figures 3A, S5A), as the incorporation of GO flakes at different concentrations had a negligible effect on the *G*′ value (Figures 3B, S5B). However, the addition of GO flakes increased the elasticity of the material, as indicated by its resistance to shear strain, since the cross points of the *G*′ and *G*″ of the amplitude sweep measurements shifted toward higher shear strain (γ%) values at higher GO concentrations (Figure 3C). It is of note that the addition of GO resulted in a faster formation of the hybrid material (within 6 h based on the vial inversion method) than the native gel, which required a longer time to fully form (at least 12 h).

**Figure 4:**
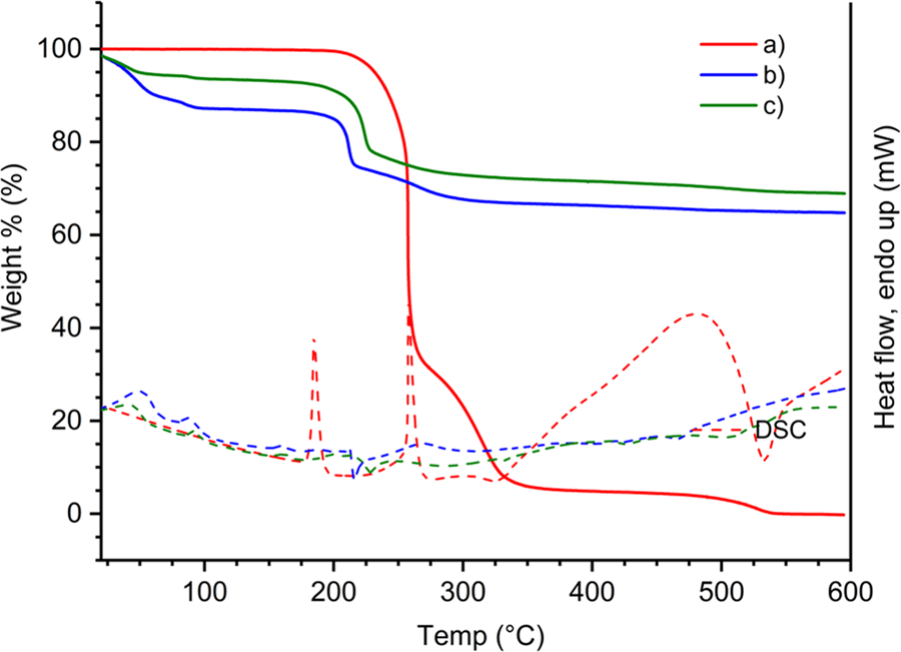
TG curves (solid lines) and DSC curves (dashed lines) of (a) neat Fmoc-F powder, (b) Fmoc-F native xerogel, and (c) Fmoc-F/GO hybrid xerogel, measured under an air atmosphere with a heating rate of 10 °C/min.

The thermogravimetric (TG) data and differential scanning calorimetric (DSC) curves of the neat Fmoc-F powder and the corresponding xerogels (native and hybrid materials) are given in Figure 4 and Table S5. The neat Fmoc-F bulk powder is free of hydrated and nonbound water as the first thermal weight loss can be observed only at 194 °C, indicating the beginning of its thermal decomposition (onset value 218 °C). The primary decomposition occurs steeply between 200 and 350 °C by various degradation and cleavage processes on the carboxylic acid and amide groups and finally at higher temperatures on the aromatic groups, resulting in a carbonaceous residue of ∼0.5 wt % at 600 °C.

On the DSC curve, the endothermic melting transition of the neat Fmoc-F powder can be seen at 184.6 °C. The TG curves of both xerogels (native and hybrid) show their first initial weight loss from 22 °C to about 100 °C, indicating the removal of residual water remaining in the xerogels (12.5 and 4.17 wt % on the native gel and hybrid material, respectively). The thermal decomposition of both xerogels initiates at a somewhat lower temperature than that of neat Fmoc-F powder, which may be due to the more porous, less structured, and highly amorphous nature of the xerogels in contrast to the highly crystalline Fmoc-F raw material. Overall, the thermal decomposition processes follow the same path in both xerogels, showing slightly higher residue on the GO-containing xerogel. This is expected due to the thermal stability of the GO sheets.

### Molecular Packing

**Figure 5:**
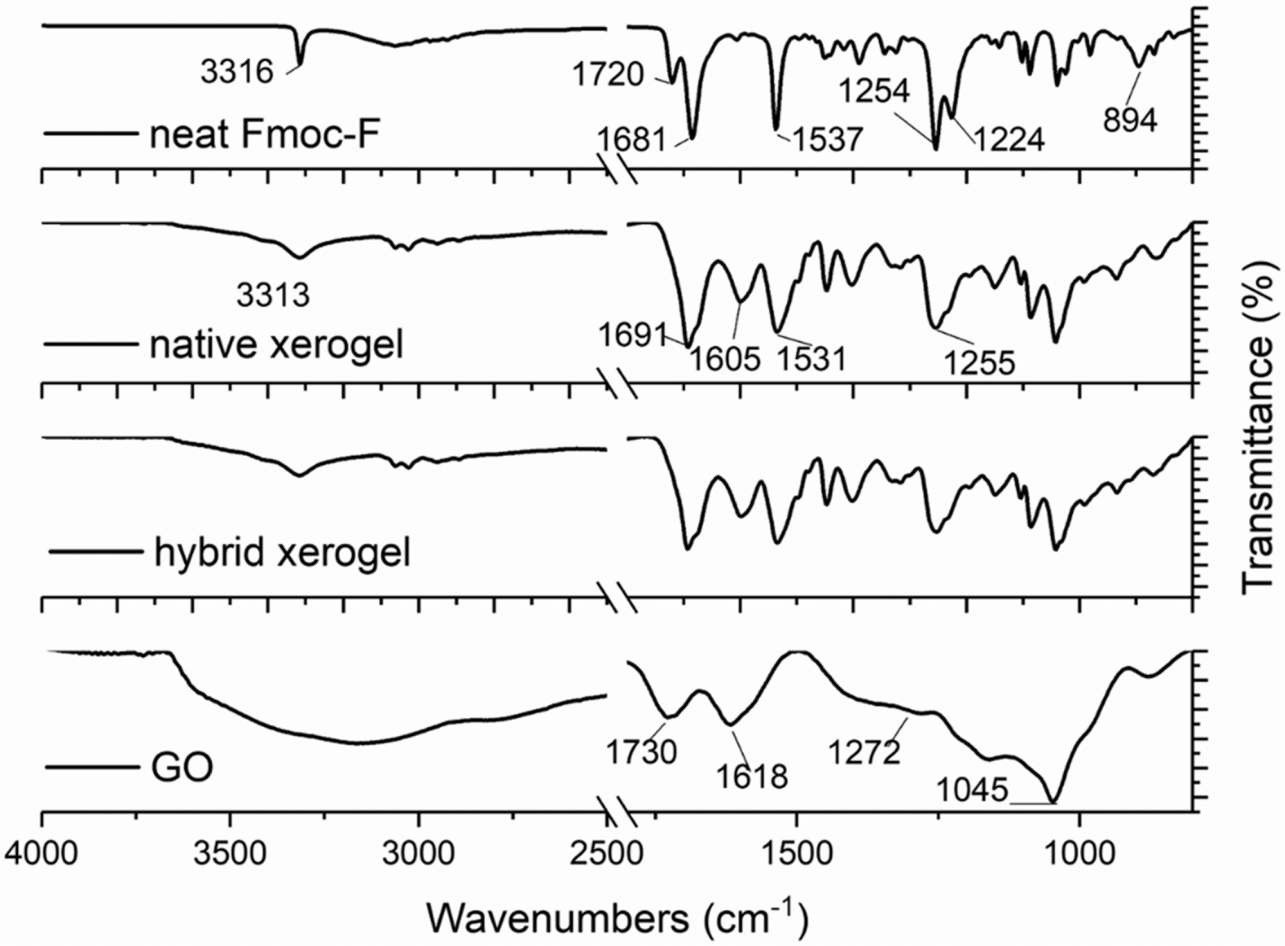
FT-IR spectra of neat Fmoc-F, neat GO, native, and hybrid xerogels. The amino acid concentration in both xerogels was 2.0 mg/mL. GO was added at a concentration of 0.25 mg/mL in the hybrid system. The FT-IR spectra of the phosphate salts Na_2_HPO_4_·2H_2_O and NaH_2_PO_4_·H_2_O used for preparing PBS solution (negative control) are given in Figure S7.

To probe the self-assembly of Fmoc-F in the presence of GO flakes, we compared the Fourier transform infrared (FT-IR) spectra of neat amino acid powder with the native (Fmoc-F) and hybrid (Fmoc-F/GO) xerogels (dried gels) and neat GO flakes (Figure 5). Both xerogels gave identical spectra, however different from neat Fmoc-F powder. Therefore, any interactions between the formed fibers and GO flakes could not be observed. The data confirm the microscopic observations that adding GO to the system did not affect the Fmoc-F self-assembly. In addition, the obtained IR profiles of both xerogels are consistent with previously reported similar systems,^33,34^ meaning that no profound changes occurred during the self-assembly of the amino acid in the hybrid system.

Both xerogels lack the 1720 cm^–1^ band of the amino acid, which corresponds to the non-hydrogen bonded carbonyl carbamate of the Fmoc group. This shows the involvement of the Fmoc moiety either in H-bond formation or other noncovalent interactions. The amide A and II bands at 3316 and 1537 cm^–1^, respectively, are shifted in both xerogels, which corroborates the formation of amide–amide H-bonding. A blue shift is also observed for the amide I band (1681 to 1691 cm^–1^). The C–O/C–N stretching peaks (1254, 1224 cm^–1^) of neat Fmoc-F merged toward a broader band in both xerogels (∼1255 cm^–1^), while the C–H out of plane band (895 cm^–1^) is diminished. Finally, neat GO gave the characteristic peaks of O–H stretching (broad ∼3430–2940 cm^–1^), C=O stretching (1730 cm^–1^), aromatic C=C and O–H bending (1618 cm^–1^), epoxy C–O stretching (1272 cm^–1^), and alkoxy C–O stretching (1045 cm^–1^), which are not seen in the hybrid xerogel.^35^

**Figure 6:**
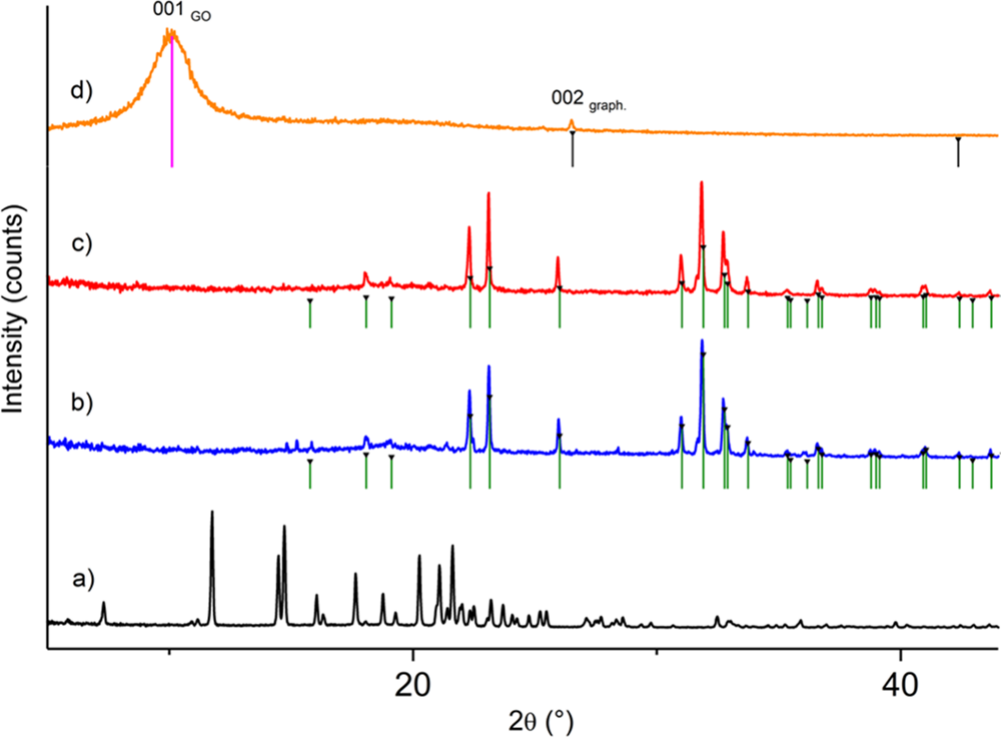
Powder X-ray diffraction patterns of (a) neat Fmoc-F, (b) Fmoc-F native xerogel, (c) Fmoc-F/GO hybrid xerogel, and (d) neat GO. Green vertical markers correspond to characteristic Bragg peak positions of anhydrous Na_2_HPO_4_, originating from the phosphate buffer solution, which crystallized in both xerogel samples. Black and magenta markers represent graphite and GO phases, respectively. The concentration of Fmoc-F was 2.0 mg/mL in both xerogel samples. GO was added at a concentration of 0.25 mg/mL in the hybrid system.

To further explore potential differences in the structure of the materials, we compared the powder X-ray diffraction (PXRD) patterns of the native and hybrid xerogels with the neat bulk powder of Fmoc-F and GO flakes (Figure 6). The Pawley fit (Figure S6) indicates that the crystalline bulk powder, with sharp, distinct diffraction peaks, is phase pure and structurally congruent with the reported single crystal structure since no unindexed peak positions remain in the fit. The crystallographic data and the agreement indices are given in Table S6.

The native Fmoc-F xerogel shows several sharp, distinct diffraction peaks at the angular range of 15–40° 2θ. However, a search-match phase identification analysis indicated that the obtained peaks do not originate from the Fmoc-F phase. Instead, they are unambiguously characteristic of the anhydrous Na_2_HPO_4_ phase. The phosphate phase originates from the buffer solution, crystallized during the drying of the hydrogel. Similar peaks have also been reported previously for the Fmoc-F xerogel (gel samples prepared in PBS solution by sonication/heating).^34^ Here, all gel samples were prepared in PBS solution with sonication/heating-induced gelation, as reported by Thakur et al.,^14^ whose materials showed antibacterial properties against Gram-positive bacteria in the solution and gel phases. To avoid strong X-ray diffraction of phosphate salts, gels could be prepared in water, and gelation triggered by the pH switch method. However, we have intentionally followed the gelation protocol of Thakur et al.^14^ to prepare materials with known antimicrobial properties. Also, changes in the gelation method and/or the solvent alter self-assembly mode resulting in materials with different properties. In addition to the phosphate phase peaks, a few very weak peak positions remain unindexed in the pattern, for example, at 14.8°, 15.3°, 19.7°, 20.0°, and 20.7° 2θ. The peaks may correspond to a small contribution of a different Fmoc-F polymorph or a hydrated form, as suggested by Singh et al.^34^ Despite the above findings, Fmoc-F in the native xerogel exists in an amorphous form.

The PXRD pattern of neat GO shows that the sample is practically amorphous, as only a few very broad diffraction peaks (humps) can be observed. The strongest broad peak at 10.08° is the characteristic carbon (001) peak for GO sheets, corresponding to a definite *d* spacing of 0.8–0.9 nm, as reported by Marcano et al. and Yasin et al.^36,37^ The GO sample contains also traces of graphite, which is best seen by the characteristic (002) peak at 26.52° 2θ.

The diffraction pattern of the hybrid xerogel is clearly reminiscent of the native Fmoc-F xerogel pattern. In both patterns, the strongest diffraction peaks can be assigned to the anhydrous Na_2_HPO_4_ phase. The most significant difference between hybrid and native xerogels is the lack of additional weaker peaks, suggesting that the Fmoc-F fibers have collapsed to a fully amorphous form during the preparation of the hybrid xerogel. This differs, for example, from the previously reported structurally similar Fmoc-glutamic acid/GO gel system, in which a weak crystalline phase was observed.^38^ It is also noted that the broad peak of GO and the weaker peak of the graphite phase are missing from the hybrid xerogel PXRD pattern. This suggests that the GO sheets most likely interact with the Fmoc-F fibrous network, which in turn partially causes some delamination of the GO sheets and, thereby, the 10.08° peak is absent.

**Figure 7:**
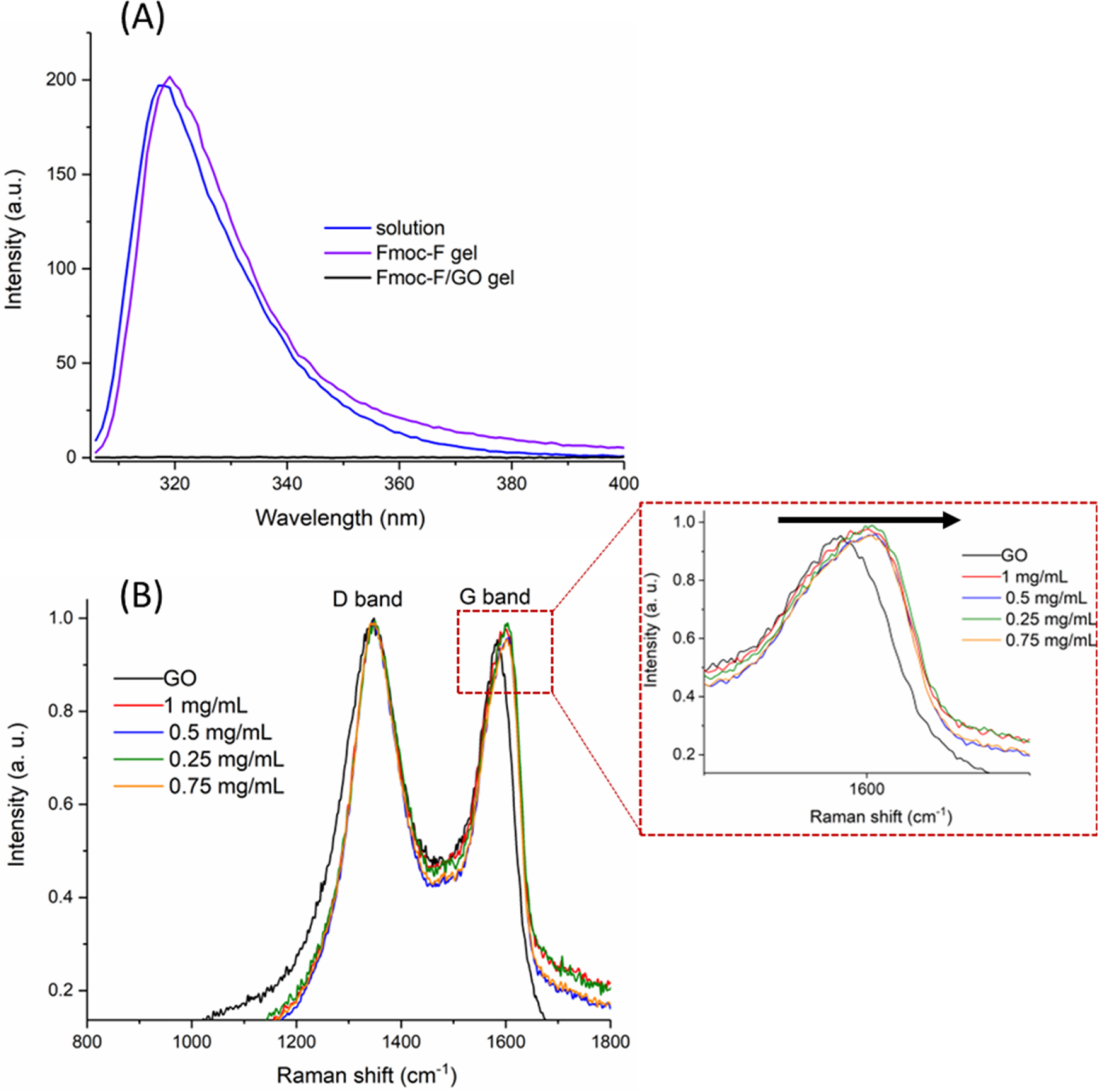
Spectroscopy analysis of the hydrogels. (A) Fluorescence spectra of the Fmoc-F solution in DMSO, native, and hybrid hydrogels. The concentration of Fmoc-F was 2.0 mg/mL and GO 0.25 mg/mL. The corresponding UV–vis spectra are given in Figure S8. (B) Raman spectra of neat GO and the hybrid hydrogel at a range of GO concentrations. The concentration of Fmoc-F was kept constant (2.0 mg/mL). The insert depicts a magnification of the G band plots with an arrow indicating the blue shift by addition of GO.

To explore potential changes in the fluorescent properties of Fmoc-F amino acid at the gel state, we compared the emission spectra of the native and hybrid hydrogels with Fmoc-F at the solution state (Figure 7A). The amino acid shows a strong emission at the solution state, centered at 318 nm on excitation at 296 nm. No significant shifting is observed at the native hydrogel (emission, 319 nm; excitation, 296 nm). However, the fluorescence emission of the hybrid material is quenched, suggesting either the development of supramolecular interactions between the amino acid (formed Fmoc-F fibers) and GO flakes or just their spatial proximity. To support our findings, we performed Raman spectroscopy studies of the hybrid hydrogel at a range of GO concentrations (Figure 7B). As expected, neat GO showed two fundamental vibrations at ∼1345 and ∼1583 cm^–1^, corresponding to the D and G bands, respectively. The D or disorder-induced band is indicative of lattice defects or appears near the edges of graphene, while the G or graphitic vibrational mode is due to the in-plane motion of the sp^2^ hybridized carbon atoms (bond stretching). For all gel samples, irrespective of the amount of added GO, the ID/IG ratio is higher than that of neat GO. In addition, the G band is blue-shifted toward higher values compared to neat GO (∼1583 to ∼1600 cm^–1^). These observations suggest a decrease in the size of the GO basal plane (in-plane sp^2^ domains), presumably due to the development of π–π interactions between the Fmoc group of the amino acid and the basal plane of GO flakes.^38^

### Antimicrobial Screening

**Figure 8:**
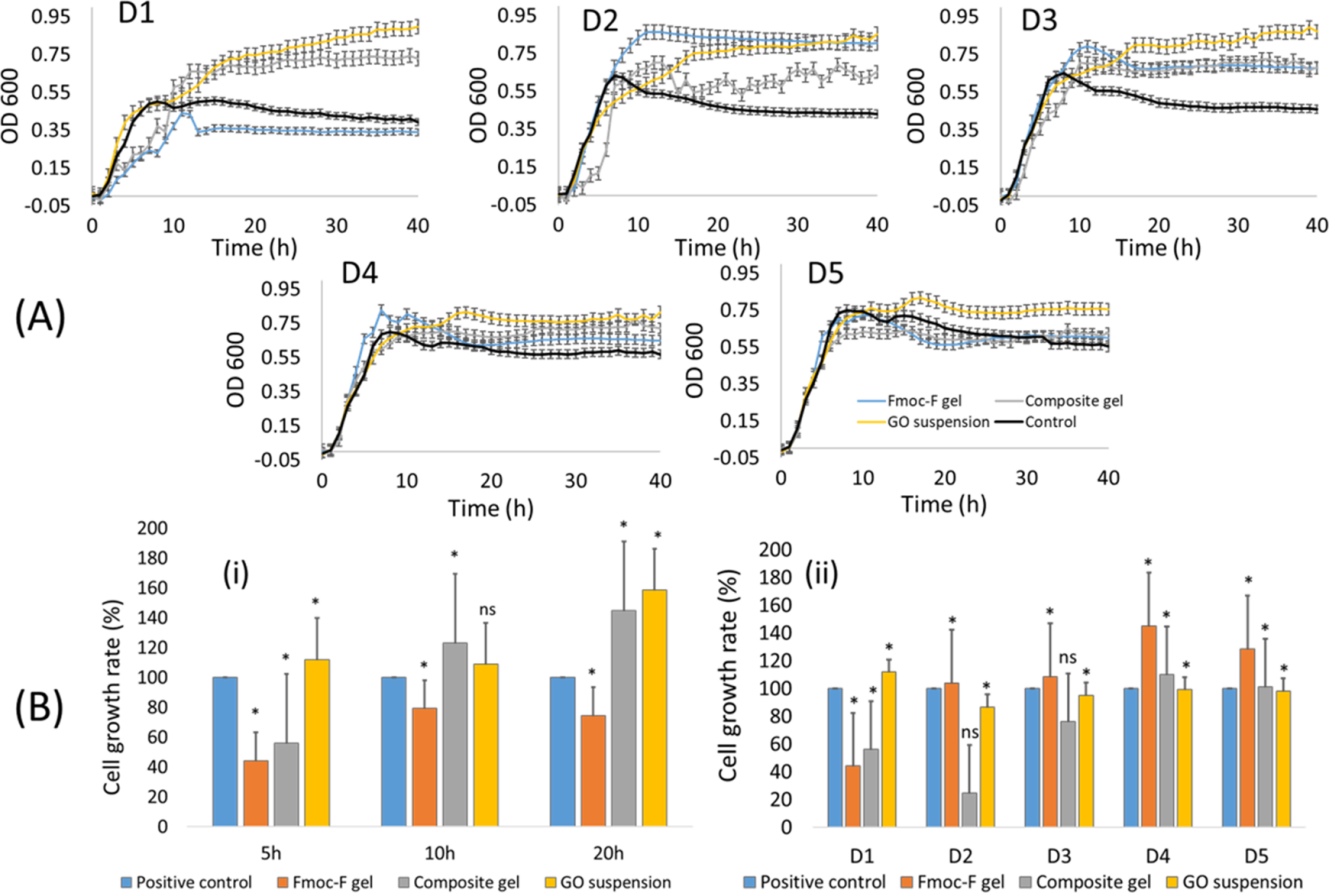
Antibacterial effect of GO flakes and native (Fmoc-F) and hybrid (Fmoc-F/GO) hydrogels against *E. coli.* (A) Optical density measurements of the treated bacterial cultures at five different concentrations over 40 h. (B) The cell viability at different time points of incubation at D1 concentration (i) and after 5 h of incubation at all five concentrations (D1–D5) (ii). *p < 0.05; ns, nonsignificant (in relation to the control); statistical analysis was performed with t-Test; *n* = 3. Error bars denote standard deviation.

**Figure 9:**
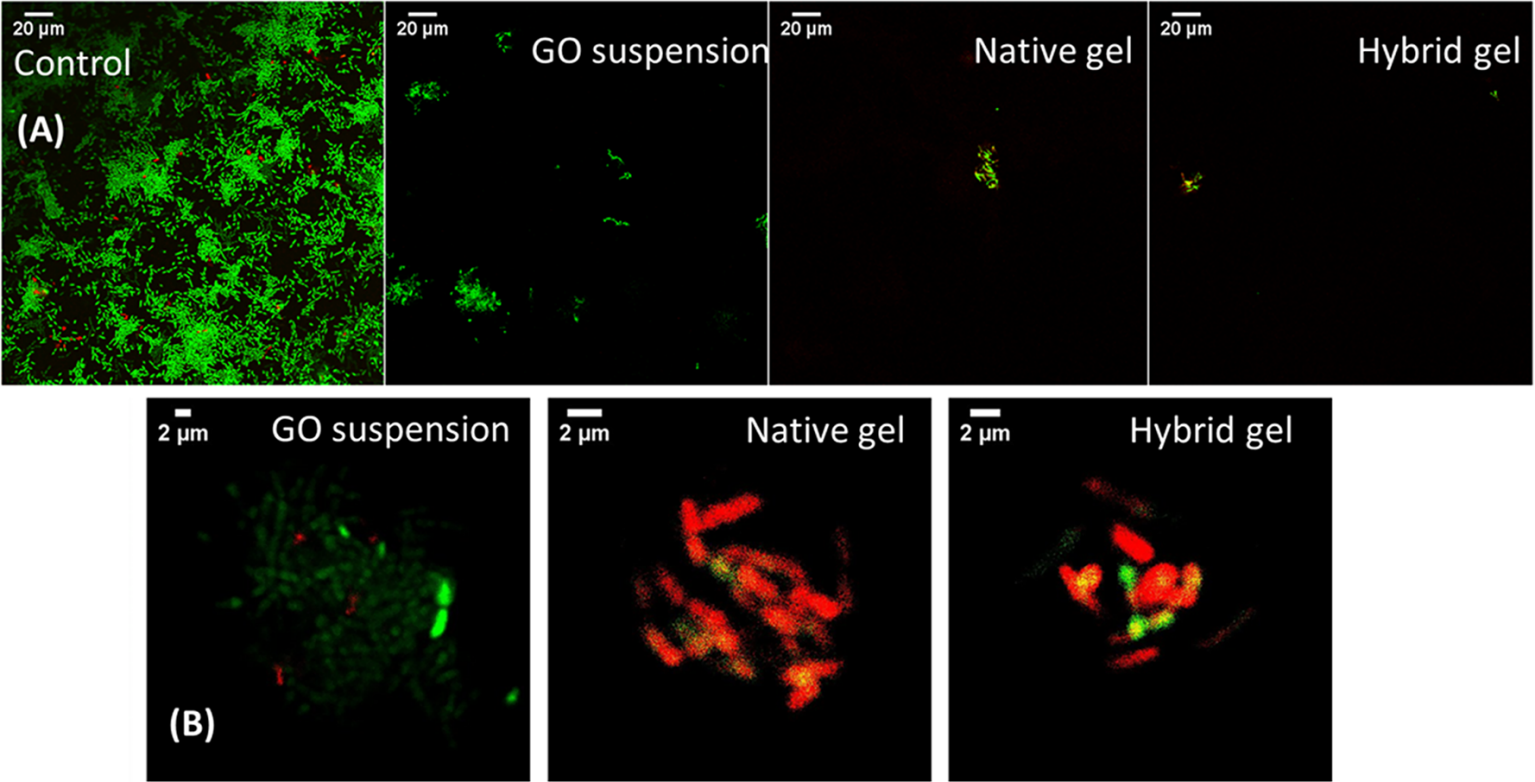
Live/dead staining images of the bacteria after 5 h of incubation at D1 concentration at two different magnifications (A and B). The green fluorescence indicates bacteria with both intact and damaged membrane/wall, and the red fluorescence indicates dead bacteria cells.

The antimicrobial activity of neat GO (suspension in PBS) and native (Fmoc-F) and hybrid (Fmoc-F/GO) hydrogels was assessed against Gram-negative *E. coli*. The bacterial growth was evaluated over time, *in vitro*, by measuring the optical density of the treated cultures at a wavelength of 600 nm (OD_600_). The results were then translated into cell growth rate (%) by considering the cell survival (OD_600_) of the untreated bacteria (control) as 100%. The samples were evaluated at five different concentrations, prepared by serial dilutions (Table 1, D1–D5) over a period of 40 h (Figure 8). In addition to bacterial growth, the integrity of the bacterial membrane/wall was further assessed by a live/dead staining assay (Figure 9, intact cells are viable cells).

The Fmoc-F native hydrogel, as expected, showed poor bactericidal efficacy, especially after the second dilution (D2). The hybrid hydrogel, however, inhibited *E. coli* growth over three consecutive dilutions (D1–D3), while the GO suspension showed negligible antimicrobial effects at all five concentrations (Figure 8B,ii). Interestingly, the most profound delay in growth population was observed during the first 5 h of incubation for both gel samples, native and hybrid, at the first dilution (Figure 8A, D1). Indeed, the cell growth rate for the native gel was 44% and for the hybrid gel was 56% compared to the control (Figure 8B,i).

After 10 h of incubation, at the first dilution (Figure 8A, D1), only the native hydrogel inhibited bacterial growth, which was kept below that of the control until the end of the measurement (40 h). At the same time point (10 h), the hybrid gel lost its inhibition effect as after that (from 10 to 40 h), the observed cell growth exceeded that of the control (Figure 8A, D1). Further dilutions of the Fmoc-F native gel (D2–D5) did not inhibit/delay the bacterial growth either, resulting in a cell growth increase (Figure 8B, ii). The hybrid hydrogel, instead, led to lower bacterial populations compared to the control for the first three consecutive dilutions (D1–D3). Notably, the inhibition/delay of the bacterial growth for dilutions D1–D3 occurred during the first 5 h of incubation with corresponding cell growth rates of 56%, 25%, and 76% at D1, D2, and D3 dilutions, respectively. After 10 h of incubation, the recorded OD_600_ values exceeded the control values, demonstrating the lack of inhibition effects and bacterial regrowth (Figure 8A, D1, D2, D3).

The data showed that the hydrogels and the GO suspension demonstrate poor antibacterial activity against Gram-negative *E. coli*. Although the bacterial growth was delayed for the first 5 h of incubation and a large number of cells died, the remaining ones developed resistance over time and managed to increase their population density compared to the untreated cells. This could be explained by the “*inoculum effect*” (IE), in which the antimicrobial outcome of a bactericidal depends on the initial population size.^39^ Several mechanisms related to the IE, such as the “*phenotypic heterogeneity*” and “*bacterial density*”, may affect the cell–hydrogel interactions. Therefore, the abrupt bacterial death (in our case within the first 5 h of incubation) is followed by the regrowth of the surviving bacteria, on which the hydrogels do not have an effect due to the IE. In addition, incorporating GO flakes in the gel system may increase the surface area upon which the bacteria can grow. Finally, the extra lipopolysaccharides at the cell wall of Gram-negative *E. coli* could protect them from the Fmoc-F hydrogel, which shows bactericidal effects against Gram-positive bacteria.^10^

To further detect the antibacterial efficacy of the samples and evaluate the integrity of the bacterial membrane/wall, we performed a live/dead staining assay, after 5 h of incubation, at the first dilution D1 (Figure 9). The green fluorescent dye (Syto9) stains both live and dead cells, in contrast to the red fluorescent dye (PI), which selectively stains bacteria with destroyed cell walls and membranes. The live/dead imaging data are only qualitative and complement the OD_600_ findings, meaning no statistical analysis was performed about the percentage of dead cells. The imaging data were consistent with the OD_600_ findings. As expected, the untreated bacteria were intact and stained mainly green, while no changes were observed in their morphology. Similarly, those treated with GO suspension were predominantly stained green, with some negligible red fluorescence also present. However, the bacteria treated with both gel samples were mainly stained red, suggesting that most cells were dead.

## Conclusions

In summary, we studied the gelation synergies of Fmoc-F amino acid and GO flakes and assessed the antimicrobial efficacy of the formed hybrid material against Gram-negative *E. coli* for the first time. GO flakes do not affect the self-assembly of Fmoc-F amino acid per se, but the formed fibers interact with the flakes, as we observed by spectroscopy analysis. The incorporation of GO flakes modulates the viscoelastic properties of the hybrid material, which also forms faster than the native gel. The hybrid hydrogel showed poor antimicrobial activity against *E. coli*, likewise the native gel, probably due to the inoculum effect. However, due to its mechanical and physicochemical properties, the Fmoc-F/GO hybrid hydrogel has a high potential for advancing the development of bactericidal soft materials, for example, via the selective immobilization of antibacterial agents on the surface area of GO flakes.

## Supporting Information Available

Concentration screening; gelation and phase transition temperature measurements; helium ion microscopy of native gel; rheological studies; thermogravimetric and differential scanning calorimetry analysis results; powder X-ray diffraction analysis results (PDF)
