# Supplementary material for: Probing the Gelation Synergies and Anti-Escherichia coli Activity of Fmoc-Phenylalanine/Graphene Oxide Hybrid Hydrogel
Source: ACS Omega. 2023 Mar 8;8(11):10225–34. doi: 10.1021/acsomega.2c07700 (PMC10034972; doi:10.1021/acsomega.2c07700)
Supplement: Supplementary file 1 — ao2c07700_si_001.pdf [file ao2c07700_si_001.pdf]

Supporting information for

Probing the gelation synergies and anti-Escherichia coli activity of Fmoc-phenylalanine/graphene oxide hybrid hydrogel

Efstratios D. Sitsanidis,[a] Lara A. L. Dutra,[b] Johanna Schirmer,[a] Romain Chevigny,[a] Manu Lahtinen,[a] Andreas Johansson,[a,c] Carmen C. Piras,[d] David K. Smith,[d] Marja Tiirola,[b] Mika Pettersson[a] and Maija Nissinen[a*]

[a.]Department of Chemistry, Nanoscience Center, University of Jyväskylä, P.O. Box 35, FI-40014 JYU, Finland.
[b.]Department of Biological and Environmental Sciences, Nanoscience Center, University of Jyväskylä, P.O. Box 35, FI-40014 JYU, Finland.
[c.] Department of Physics, Nanoscience Center, University of Jyväskylä, P.O. Box 35, FI-40014 JYU, Finland.
[d.] Department of Chemistry, University of York, Heslington, York, YO10 5DD, UK.

Table of Content

1. Concentration screening 2
2. Gelation and phase transition temperature measurements 3
3. Helium Ion Microscopy 5
4. Rheological studies 6
5. Thermogravimetric and differential scanning calorimetry analysis 7
6. Powder X-ray diffraction 8

References 9

1. Concentration screening

Table S1. Concentration screening trials of the Fmoc-F native hydrogel and corresponding phase transition temperature measurements

Fmoc-F (mg/mL)	Gelation outcome	$T_{gel-sol}$ (°C)
1.0	No	-
2.0*	Yes	35
3.0	Yes	50
4.0	Yes	55
5.0	Yes	60
6.0	Yes	60

*Critical gelation concentration

Table S2. Concentration screening trials of the GO flakes on the critical gelation concentration of Fmoc-F

Fmoc-F (mg/mL)	Gelation without GO	GO (mg/mL)	Gelation with GO
1.0	No	0.25	No
		0.5	No
		0.75	No
		1.0	No
2.0*	Yes	0.25	Yes
		0.5	Yes
		0.75	Yes
		1.0	Yes

*Critical gelation concentration of Fmoc-F.

2. Gelation and phase transition temperature measurements

Table S3. Gelation trials and phase transition temperature measurements of the Fmoc-F/GO hybrid hydrogels. Gelation occurred under constant Fmoc-F concertation (2.0 mg/mL)

GO (mg/mL)	Gelation	Tgel-sol (℃)
0.25	Yes	40-45
0.5	Yes	40-45
0.75	Yes	50
1.0	Yes	50

Table S4. Gelation trials and phase transition temperature measurements of the Fmoc-F/GO hybrid hydrogels. Gelation occurred under constant Fmoc-F concertation (6.0 mg/ mL)

GO (mg/mL)	Gelation	Tgel-sol (℃)
0.25	Yes	60
0.5	Yes	60
0.75	Yes	60
1.0	Yes	60

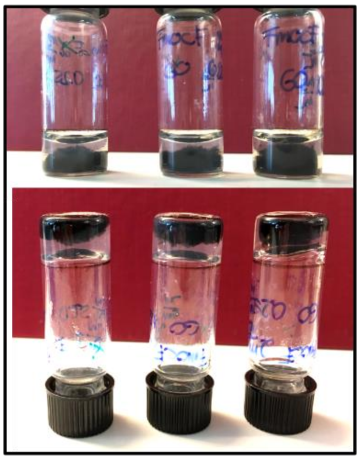

Figure S1. Heating the Fmoc-F/GO suspension at 95 ℃ for 1 h led to the precipitation of GO (gel sample in triplicate). The concentration of Fmoc-F was 2.0 mg/mL, and that of GO flakes was 0.25 mg/mL.

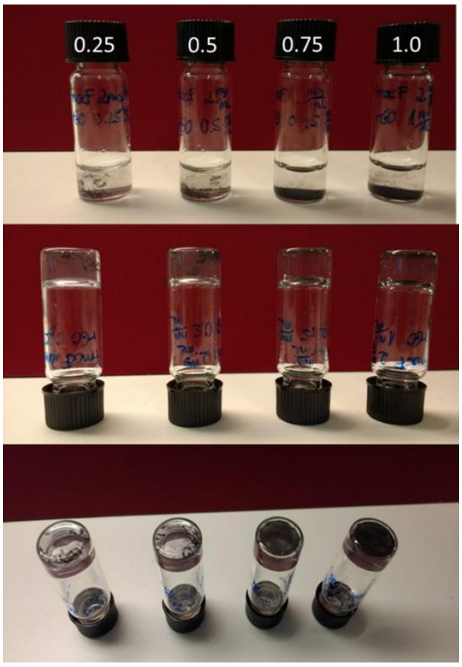

Figure S2. Gelation screening of the Fmoc-F/rGO hybrid system at a range of rGO concentrations (mg/mL). The precipitation of rGO was observed irrespective of its concentration. The concentration of Fmoc-F was constant (2.0 mg/mL).

3. Helium Ion Microscopy

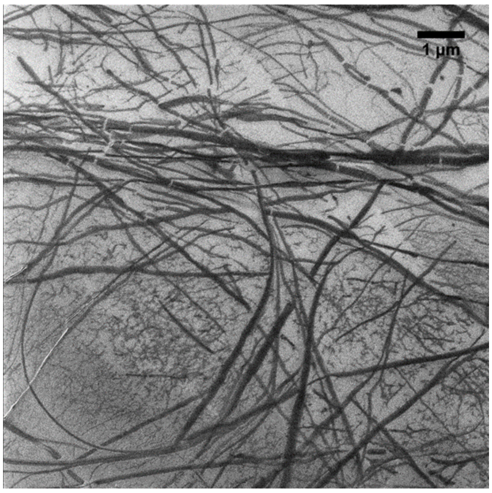

Figure S3. Helium ion microscopy image of the native Fmoc-F hydrogel. The formed fibres are similar to those of the hybrid Fmoc-F/GO material. The concentration of Fmoc-F was 2.0 mg/mL.

4. Rheological studies

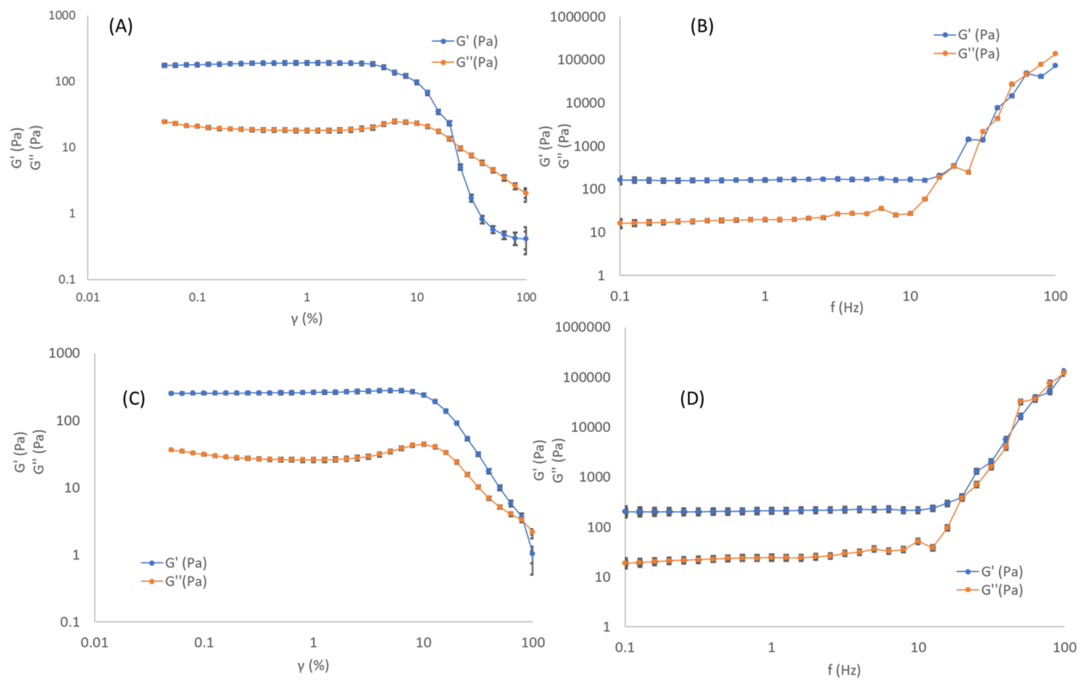

Figure S4. Rheology studies. Amplitude (A) and frequency sweep (B) measurements of the native hydrogel. Amplitude (C) and frequency sweep (D) measurements of the hybrid gel. The concentration of Fmoc-F was 2.0 mg/mL for both samples and GO 0.25 mg/mL for the hybrid gel. Amplitude sweep measurements were performed at an angular frequency of 1.0 Hz, using shear strain (γ%) within the range of 0.05% - 100%. Frequency sweep measurements were performed in triplicates using a shear strain (γ%) of 0.25%, at a range of 0.1 to 100 rad/s. All measurements were performed at 25 °C. Error bars denote the standard deviation.

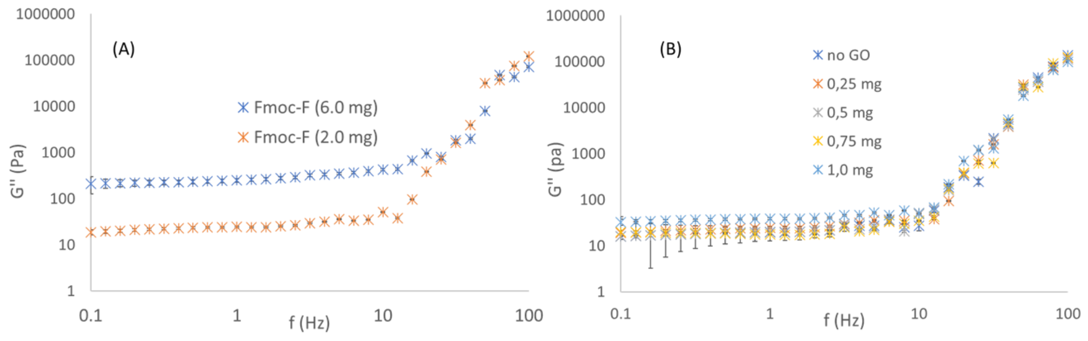

Figure S5. (A) Corresponding loss moduli (G´´) values of Figures 3A and 3B. (A) The effect of Fmoc-F concentration on G´´;(B) The effect of GO addition on G´´. Frequency sweep measurements were performed in triplicates using a shear strain (γ%) of 0.25%, at a range of 0.1 to 100 rad/s. All measurements were performed at 25 °C. Error bars denote the standard deviation.

5. Thermogravimetric and differential scanning calorimetry analysis

Table S5. The summary of TG/DSC analysis results

**Sample**	**Weight loss per step (wt.- %), Temp. range (°C)**	**Residual weight (wt.- %), Temp. (°C)**	***T_d* (°C)**	**Transitions by DSC (°C), (J/g)**
Fmoc-F neat	67.73, (194 - 272)<br>26.61, (272 - 373)<br>5.15, (373 - 545)	0, 545	218	184.6, (128.06)
Fmoc-F xerogel	12.50, (20 - 102)<br>12.79, (102 - 221)<br>7.10, (221 - 309)<br>2.59, (309 - 595)	64.78*, 595	200	159.0, (6.68)
Fmoc-F/GO xerogel	4.17, (20 - 104)<br>16.84, (104 - 233)<br>6.14, (233 - 333)<br>6.14, (333 - 595)	68.49*, 595	200	159.8, (6.02)

* mainly phosphate salts.

7. Powder X-ray diffraction

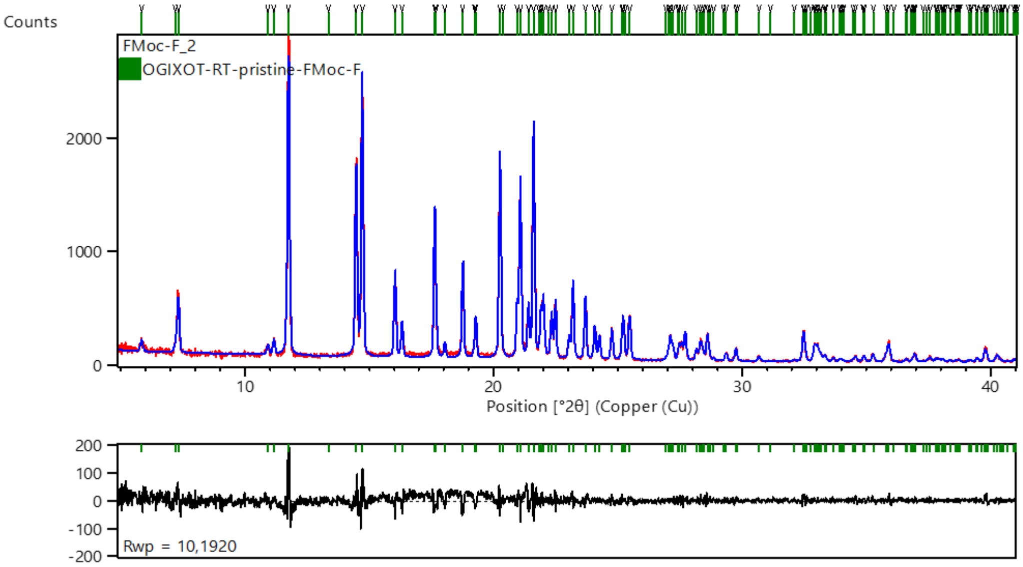

Figure S6. Pawley refinement plot of Fmoc-F. The experimental pattern is shown in red, the fitted profile in blue, and the green-coloured vertical markers correspond to the Bragg peak positions of the indexed unit cell. The difference plot of the experimental vs refined profile is shown below in black.

Table S6. Crystallographic data of Fmoc-F measured by powder and single-crystal diffraction[1]

**Parameters**	**PXRD**	**SC-XRD[1]**
Temp [°C]	22	-173
Crystal system	monoclinic	monoclinic
Space group	$P2_1$	$P2_1$
a /Å	13.206(2)	13.1570(13)
b /Å	4.9637(8)	4.9083(4)
c /Å	16.234(2)	16.1242(16)
α /°	90	90
β /°	112,712(1)	113.135(3)
γ /°	90	90
V/Å^3	981,65(23)	957.54(16)
$R_{exp.}$	0.0891	
$R_{prof.}$	0.0740	
$R_{w\text{-}prof}$	0.1019	
R_1		0.0455
wR_2		0.1027
GOF	1.14327	0.996

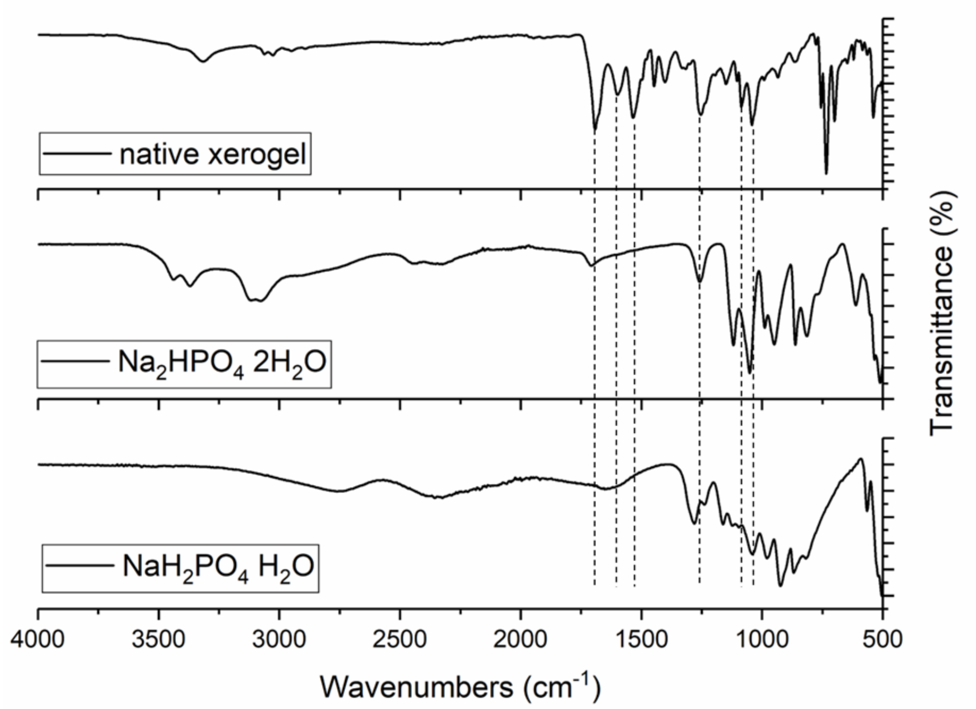

Figure S7. The FT-IR spectra of the native xerogel (Fmoc-F prepared in PBS solution) and phosphate salts $Na_2HPO_4 \cdot 2\ H_2O$ and $NaH_2PO_4 \cdot H_2O$ used for the preparation of the PBS solution (50 mM, pH 7.4, negative control). The concentration of Fmoc-F was 2 mg/mL.

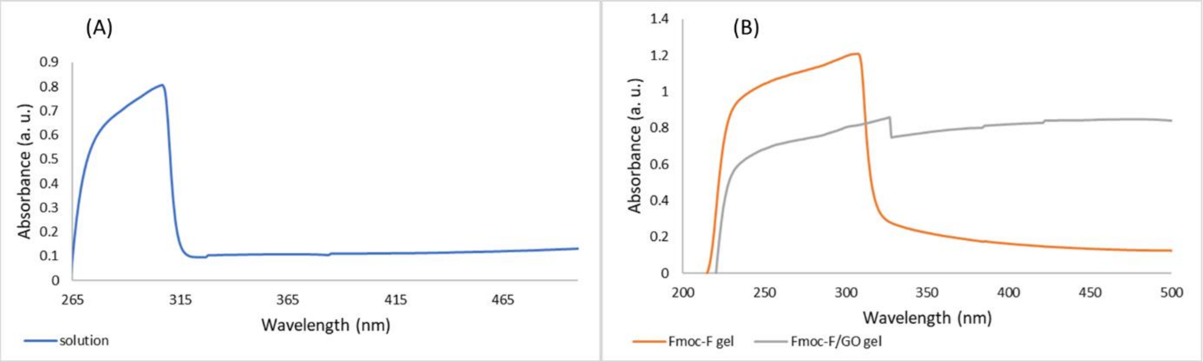

Figure S8. The UV-vis spectra of (A) the Fmoc-F solution in DMSO (spectrum cut off at 265 nm); (B) the Fmoc-F native and Fmoc-F/GO hybrid hydrogels (spectrum cut off at 200 nm). The concentration of Fmoc-F was 2.0 mg/mL and GO 0.25 mg/mL. Both gel samples were formed in situ in a quartz cuvette with a path length of 1 cm.

References

1. Draper, E. R.; Morris, K. L.; Little, M. A.; Raeburn, J.; Colquhoun, C.; Cross, E. R.; McDonald, T. O.; Serpell, L. C.; Adams, D. J. Hydrogels formed from Fmoc amino acids. *CrystEngComm.* **2015**, 17, 8047-8057.
